# Integrating Targeted Therapies into AML Frontline Therapy: Who Gets What and What Does the Future Hold?

**DOI:** 10.3390/cancers18061034

**Published:** 2026-03-23

**Authors:** Johanna Schreiber, Georg Hopfinger, Karoline V. Gleixner

**Affiliations:** 1 Department of Internal Medicine III, Division of Hematology and Oncology, Klinik Favoriten, 1100 Vienna, Austria; georg.hopfinger@gesundheitsverbund.at; 2 Association for the Promotion of Medical Education and Research in Vienna-Favoriten, Bahnhofplatz 6, 2340 Moedling, Austria; 3 Paracelsus Medical University, 5020 Salzburg, Austria; 4 Department of Internal Medicine I, Division of Hematology and Hemostaseology, Medical University of Vienna, 1090 Vienna, Austria; karoline.gleixner@meduniwien.ac.at

**Keywords:** acute myeloid leukemia, targeted therapy, FLT3, IDH1, IDH2, venetoclax, menin inhibitors, KMT2A, frontline treatment, precision medicine

## Abstract

For decades, induction treatment of acute myeloid leukemia consisted of intensive chemotherapy for induction. High relapse rates and severe toxicity resulted in a five-year overall survival of ~30%. In patients ineligible for intensive treatment, hypomethylating agents (HMA) could be administered but generally failed to induce durable remissions. These limitations have driven the development of targeted drugs and less toxic therapeutic regimens. In the past decade, fourteen new agents have gained FDA and/or EMA approval, including small-molecule inhibitors targeting FLT3, IDH1, IDH2, BCL-2, menin, and the hedgehog pathway, as well as a CD33-directed antibody-drug conjugate. The combination of targeted drugs with intensive chemotherapy or HMA has resulted in improved remission rates and prolonged survival in certain patient subpopulations. However, many promising combinations are currently being evaluated in randomized trials and are not yet available in clinical routine. A combination that has become standard of care is HMA plus venetoclax for patients unfit for intensive chemotherapy, achieving high remission rates with relatively manageable toxicity. Moreover, targeted drugs directed against FLT3 and IDH1 have been approved in combination with intensive chemotherapy and HMA, respectively. Clinical decision-making requires rapid molecular diagnostic testing, assessment of a patient’s fitness for intensive chemotherapy, and management of toxicities and drug interactions. This narrative review, illustrated with patient vignettes, summarizes currently available therapies, guides through the latest trials on frontline combinations in AML, and provides a preview of how the therapeutic landscape may evolve in the near future.

## Simple Summary

Acute myeloid leukemia is an aggressive blood cancer that, for decades, was treated with intensive chemotherapy alone, resulting in poor long-term survival for most patients due to relapse and toxicity. In recent years, a growing number of drugs targeting specific genetic changes in leukemic cells have been approved, offering more personalized treatment options. However, the rapid expansion of available therapies has created new challenges for clinicians: determining which drug is best suited for which patient, how to combine these agents with existing treatments, and how to manage their side effects. This review provides a practical guide for integrating targeted therapies into the frontline treatment of acute myeloid leukemia. Using illustrative patient cases, we discuss current evidence for each major molecular subgroup, highlight promising drug combinations under investigation, and outline strategies for treatment selection in everyday clinical practice.

## 1. Introduction

For decades, the standard treatment for acute myeloid leukemia (AML) has been the combination of cytarabine and anthracycline, commonly known as the 7+3 regimen [1,2]. Whole genome sequencing has dramatically accelerated progress in translational clinical research, enabling the discovery of driver mutations and the development of targeted therapy [3].

Since 2017, 13 agents have been approved for AML by the FDA and 10 by the EMA. These approvals include venetoclax [4,5], gemtuzumab ozogamicin [6], three FLT3 inhibitors (midostaurin [7], gilteritinib [8], and quizartinib [9]), three IDH inhibitors (ivosidenib [10,11], enasidenib [12], and olutasidenib [13]), two oral hypomethylating agents (decitabine-cedazuridine [14] and oral azacitidine (CC-486) [15]), the liposomal formulation of cytarabine and daunorubicin (CPX-351) [16], the hedgehog inhibitor glasdegib [17], and recently, the menin inhibitors revumenib [18,19] and ziftomenib [20] (Table 1). This rapid expansion has created uncertainty about how to optimally integrate these agents into front-line therapy. Traditional regimens such as 7+3 were designed for broad patient populations, but this one-size-fits-all approach fails to address the molecular heterogeneity of AML. Beyond these approved agents, a growing number of novel combination strategies are under active investigation in clinical trials (Table 2). We therefore address the questions of which targeted therapies are currently available for which patients, how they can be combined with intensive chemotherapy (IC) or hypomethylating agents (HMA), and which important research directions are being pursued.

Herein, we present a series of patient cases that reflect AML’s biological and clinical heterogeneity. For each scenario, we outline a treatment strategy integrating targeted agents into the current therapeutic landscape and discuss remaining challenges in treatment selection.

## 2. FLT3-Mutated AML—Integrating FLT3 Inhibitors with Chemotherapy and Low-Intensity Therapy

A fit 68-year-old, otherwise healthy, woman presented to the emergency department with a swollen right leg and was found to have a deep vein thrombosis. Laboratory tests revealed a white blood count of 175.10 × 10^9^/L with ~43% circulating blasts, hemoglobin 7 G/dL, and platelets 21.10 × 10^9^/L. Bone marrow aspiration and biopsy confirmed the diagnosis of AML (FAB myelomonocytic M4) with 80% blasts. Molecular profiling revealed an *FLT3* internal tandem duplication (*FLT3*-ITD) (~50% variant allele frequency (VAF)) and a *DNMT3A* (~48% VAF) mutation. No mutations in *NPM1*, *IDH1/2*, or *TP53* were detected. Cytogenetics showed a normal karyotype. By international consensus classification (ICC) 2022, her disease was classified as AML, not otherwise specified (AML-NOS), and her genetic profile placed her in an intermediate risk by ELN 2022 [53].

### 2.1. What Can We Offer This Patient?

#### 2.1.1. FLT3 Inhibitors for IC-Eligible Patients

Standard induction for fit patients is 7+3 chemotherapy. In the frontline setting, two FLT3 inhibitors are available in combination with IC: midostaurin, studied in the RATIFY trial (*FLT3*-ITD or tyrosine kinase domain (TKD), age < 60), and quizartinib, evaluated in the QuANTUM-First trial (*FLT3*-ITD, age 18–75) [7,9]. In a relapsed/refractory (R/R) setting, single-agent gilteritinib has demonstrated superior response rates, complete remission/complete remission with partial hematologic recovery (CR/CRh) (34% vs. 15%), and a median overall survival (mOS) of 9.3 vs. 5.6 months compared to salvage chemotherapy in the ADMIRAL trial [8]. However, gilteritinib is not available for first-line treatment so far. In the frontline several factors should be considered when choosing between midostaurin and quizartinib:

**Mutation subtype and selectivity**: FLT3 inhibitors are classified by their binding mode: type 1 inhibitors (e.g., midostaurin and gilteritinib) bind the FLT3 ATP pocket in both active and inactive conformations and inhibit both ITD and TKD mutations, whereas type 2 inhibitors (e.g., sorafenib and quizartinib) bind only the inactive conformation and only affect ITD-type mutations [54]. In our patient with an *FLT3*-ITD mutation, either drug could be used. If this were an *FLT3*-TKD mutation, midostaurin would be the clear choice. However, in the RATIFY subgroup analysis, patients with *FLT3*-TKD mutations did not derive a statistically significant overall survival (OS) benefit from midostaurin (HR 0.65, 95% CI 0.39–1.08), suggesting that TKD mutations may be less sensitive to midostaurin-based therapy [7]. Quizartinib is a potent and highly selective FLT3 inhibitor, targeting primarily FLT3 and achieving deeper FLT3 inhibition, whereas midostaurin is a multi-kinase inhibitor that hits FLT3 and other kinases (e.g., KIT and PDGFR) [55]. There has been no head-to-head trial of midostaurin vs. quizartinib so far.

**Patient Age and Sex:** Our patient is 68 years old. Age is an important consideration, since the FLT3 inhibitor trials had different age populations and efficacy patterns by age. In the phase 3 RATIFY trial (CALGB 10603), the addition of midostaurin (days 8–21) during induction and consolidation, plus 12 months of maintenance, led to a mOS of 74.7 vs. 25.6 months with placebo [7]. This led to approval of midostaurin in newly diagnosed (ND) *FLT3*-mutated (*FLT3*m) AML by FDA/EMA. RATIFY did not enroll patients ≥ 60. However, the AMLSG 16-10 trial (single-arm midostaurin plus 7+3) that enrolled patients aged 61–70 showed similar outcomes relative to historical controls: the composite complete remission (CRc) rate was ~72% and mOS was ~22.7 vs. 8.4 months in matched controls [56]. The phase 3 QuANTUM-First trial included patients up to age 75 (40% of participants were ≥60). Quizartinib was given on days 8–21 (induction) or days 6–19 (consolidation) and had a longer maintenance (3 years) compared to midostaurin (one year). Adding quizartinib did not improve the CR rate (55% in both arms) but improved OS significantly (mOS, 31.9 vs. 15.1 months). Subgroup analyses, however, revealed an age interaction. Younger patients (<60) clearly benefited, whereas in patients ≥ 60 the survival benefit was less pronounced and not statistically significant [9]. Key efficacy and safety data for approved FLT3 inhibitors are summarized in Table 1. Notably, post-hoc sex-stratified analyses across RATIFY, QuANTUM-First, and ADMIRAL suggest differential FLT3 inhibitor efficacy by sex. In RATIFY, the OS benefit was predominantly driven by male patients (HR 0.43), whereas females showed no benefit (HR 1.42), though these findings remain exploratory and do not yet alter treatment recommendations [8,57].

**Consolidation and Maintenance**: Consolidation after IC typically consists of cytarabine-based chemotherapy given with the same FLT3 inhibitor (midostaurin or quizartinib) used during induction. For patients not proceeding to allogeneic hematopoietic stem cell transplantation (allo-SCT), maintenance can be delivered as single-agent midostaurin or quizartinib after consolidation or as **oral azacitidine** (CC-486) maintenance therapy. Oral azacitidine maintenance is approved for patients in first remission after IC who are not transplant candidates, based on the QUAZAR AML-001 trial (mOS 24.7 vs. 14.8 months). In the *FLT3*m subgroup (*n* = 66), oral azacitidine improved relapse-free survival (RFS) and showed a trend toward improved OS [15]. After allo-SCT in *FLT3*-ITD AML, randomized data from the SORMAIN trial support sorafenib maintenance to reduce relapse and improve survival [58], whereas continuation of quizartinib after allo-SCT can be considered in patients who received quizartinib pre-transplant (allowed per EMA), although this indication is not included in the FDA label. In the MORPHO trial, gilteritinib maintenance missed the primary RFS endpoint overall but showed benefit in patients with detectable peri-transplant *FLT3*-ITD minimal residual disease (MRD). Among patients ≥ 60 years, gilteritinib reduced relapse regardless of MRD status but did not improve RFS or OS, possibly owing to higher non-relapse mortality [59,60]. Importantly, neither RATIFY nor QuANTUM-First included a second randomization for the maintenance phase; therefore, the independent contribution of maintenance to the observed survival benefit cannot be established with certainty [7,9].

**Safety profile**: A common side effect for midostaurin includes moderate gastrointestinal (GI) symptoms (nausea, vomiting, and diarrhea) and rash. Quizartinib tends to cause more myelosuppression and QTc prolongation.

#### 2.1.2. FLT3 Inhibitors for IC-Ineligible Patients

The added value of FLT3 inhibitors within lower-intensity regimens for unfit AML patients remains unproven in randomized trials. In LACEWING (gilteritinib + azacitidine vs. azacitidine alone in *FLT3*m AML ineligible for IC), the CRc was higher with the combination, but CR and OS were similar (9.8 vs. 8.9 months) between arms [61]. Evidence for other FLT3 inhibitors (e.g., sorafenib) combined with HMAs is still limited [62].

### 2.2. What Happened to Our Patient?

Given our patient’s intermediate-risk genetics (*FLT3*-ITD, *DNMT3A*) and her age of 68, she was started on IC with 7+3 plus midostaurin, based on the AMLSG 16-10 data supporting midostaurin in patients aged 61–70 [56]. Allo-SCT in first CR was planned, and a donor search was initiated. Unfortunately, after induction, bone marrow assessment showed refractory disease: ~40% persistent blasts in the marrow. Given the inadequate response, the patient was switched to gilteritinib. After two cycles of gilteritinib monotherapy, bone marrow blasts decreased to 20%. However, molecular profiling revealed rising VAFs for *DNMT3A* and a newly emerging *NRAS* mutation, while *FLT3*-ITD decreased to a VAF of 2.1%, suggesting clonal evolution under FLT3-selective pressure. Although gilteritinib monotherapy may require up to 6 cycles before CR is achieved [63,64], and early discontinuation is generally not encouraged, allo-SCT could not yet be performed as no donor was available. To reduce the risk of further clonal progression, we escalated therapy to an off-label triplet approach by adding azacitidine/venetoclax (AZA/VEN) to gilteritinib.

After one cycle of the triplet, the patient achieved a CR with hematologic recovery. *FLT3*-ITD became undetectable by high-sensitivity Polymerase Chain Reaction (PCR). Only minimal residual *DNMT3A* and *NRAS* mutant clones persisted by Next-Generation Sequencing (NGS). Given her favorable performance status and the availability of a matched donor, she proceeded to an allo-SCT from a matched donor with curative intent. Post-transplant recovery was uncomplicated; she had full donor chimerism and no measurable residual disease (MRD). Post-transplant FLT3 inhibitor maintenance was planned; however, unfortunately, 2.5 months post-transplant, she relapsed, now without *FLT3*-ITD but with a new *NUP98::NSD1* fusion and *WT1/NRAS* mutations. This case illustrates clonal evolution under FLT3-selective pressure, where elimination of *FLT3*-ITD clones may permit expansion of subclones harboring alternative drivers such as RAS-pathway mutations. RAS-pathway mutations, particularly in *NRAS*, represent the most common mechanism of secondary resistance to selective FLT3 inhibitors, emerging in approximately one-third of patients who progress on gilteritinib [65]. While direct RAS inhibitors are not yet clinically available for AML, multi-selective RAS inhibitors and mitogen-activated protein kinase kinase (MEK) inhibitors are under preclinical and early clinical investigation as strategies to overcome this resistance mechanism [66]. Given *NUP98* fusions’ dependence on the menin-KMT2A axis, emerging therapeutic options for such scenarios include menin inhibitors currently under investigation in clinical trials [39,67].

### 2.3. Emerging Frontline Combinations

#### 2.3.1. FLT3 Inhibitors + HMA + Venetoclax

Current efforts to improve outcomes in FLT3m AML focus on triplet therapy with HMA + venetoclax plus an FLT3 inhibitor. In a single-center pilot cohort and the multicenter phase 1/2 VICEROY study, AZA/VEN plus gilteritinib in induction-ineligible ND-FLT3m AML achieved very high CRc rates (~90–100%) and encouraging survival (mOS 29.7 months and 23 months, respectively) [25,68]. Similar efficacy has also been reported with the triplet regimen of decitabine, quizartinib, and venetoclax. However, the results of randomized trials comparing these triplet regimens to AZA/VEN alone are still awaited [25,26,69]. It is also of note that hematologic toxicity is increased by the addition of an FLT3 inhibitor to AZA/VEN. Furthermore, due to the promising first results, AZA/VEN combinations may in the future also be applied in IC-fit patients [70].

#### 2.3.2. FLT3 Inhibitors + GO + IC Combinations

Gemtuzumab ozogamicin (GO) with 7+3 is approved for CD33+ AML based on ALFA-0701 and meta-analyses showing an OS benefit in favorable- and intermediate-risk disease [6,71]. The benefit of adding GO to intensive regimens (e.g., FLAG-IDA) and to other targeted agents remains an important research question, with the potential risk of increased myelosuppression-related complications [72]. The AML19 Midotarg substudy added midostaurin to the GO + 7+3 backbone in patients with *FLT3*-ITD+ AML, demonstrating a CR/CR with incomplete hematological recovery (CRi) rate of 91%, with a 60 day mortality of 0% and a 2-year OS of 77%, and a deep MRD clearance [27]. This approach is currently being tested in the phase 3 OPTIMISE-FLT3 trial, which compares DA–midostaurin with DA–GO–midostaurin and FLAG-IDA–GO–midostaurin [73].

#### 2.3.3. Head-to-Head FLT3 Inhibitor Trials and Novel Agents

Gilteritinib is being evaluated as the frontline FLT3 inhibitor vs. midostaurin added to IC in the phase 2 PrECOG 0905 trial, showing higher CRc and transplant rates with gilteritinib but no improvement in MRD-negative rates post-induction [29]. More recently, the randomized phase 3 PASHA trial (NCT04027309) reported that gilteritinib did not demonstrate superior OS compared to midostaurin-based treatment, with comparable safety profiles. A full evaluation of secondary endpoints, including MRD data and subgroup analyses, is awaited. Crenolanib, a novel type 1 FLT3 inhibitor, achieved a CRc of 86% with 7+3 in a phase 2 study and is being compared with midostaurin in the randomized phase 3 ARO-021 trial (NCT03258931) [28]. Long-term results from these studies will help to define the optimal integration of FLT3 inhibition across frontline therapy, transplantation, and post-remission maintenance.

## 3. IDH Inhibitors: When to Incorporate IDH Inhibitors?

A 72-year-old man with chronic kidney disease and prior stroke presented with progressive fatigue. Laboratory evaluation revealed pancytopenia, and bone marrow biopsy confirmed AML with 45% blasts. Molecular profiling identified an *IDH1* mutation (42% VAF), *SRSF2* (38% VAF), and *ASXL1* (35% VAF). Cytogenetics showed a normal karyotype. The presence of *SRSF2* and *ASXL1* mutations—both WHO 2022-defining myelodysplasia-related changes—classified his disease as AML myelodysplasia-related (AML-MR). Given his comorbidities and Hematopoietic Cell Transplantation-specific Comorbidity Index (HCT-CI) score of 4, he was ineligible for IC.

### 3.1. Which Frontline Regimen Should This Patient Receive?

#### IDH-Mutated AML: Biology and Therapeutic Targeting

Isocitrate dehydrogenase mutations occur in approximately 6–16% (*IDH1*) and 8–19% (*IDH2*) of adult AML [74]. Mutant IDH enzymes generate the oncometabolite 2-hydroxyglutarate (2-HG), which competitively inhibits α-ketoglutarate–dependent dioxygenases, leading to hypermethylation and impaired hematopoietic differentiation [75]. Three oral IDH inhibitors are FDA approved: ivosidenib and olutasidenib for *IDH1*-mutated (*IDH1*m), and enasidenib for *IDH2*-mutated (*IDH2*m) AML. In the EU, ivosidenib is the only EMA-approved IDH inhibitor for AML [10]. These agents lower 2-HG and promote blast differentiation [11,13,75,76].

***IDH1*****-Mutated AML:** Based on the phase 1 AG120-C-001 trial, **ivosidenib** was first approved by the FDA in 2018 as monotherapy for R/R-*IDH1*m AML, demonstrating CR/CRh rates of 30% [11]. In the phase 3 AGILE trial in ND-*IDH1*m AML ineligible for intensive induction, long-term follow-up showed a mOS of 29.3 vs. 7.9 months with ivosidenib/azacitidine (IVO/AZA) vs. placebo/AZA (Table 1). These data supported FDA approval of IVO/AZA for ND-*IDH1*m AML ineligible for intensive induction (May 2022), followed by the EMA approval (May 2023) [10,21]. In a pooled analysis of *IDH1/2*m patients treated with AZA/VEN across VIALE-A and a prior phase 1b study, mOS was 15.2 months for *IDH1*m AML (vs. 2.2 months with azacitidine alone) [77]. Small retrospective analyses demonstrate that frontline IDH inhibitor therapy preserved the ability to salvage with venetoclax at relapse in 88% of patients (7/8), whereas only 56% (10/18) responded to IDH inhibitors after upfront venetoclax [78]. The ongoing I-DATA study (NCT05401097) is prospectively comparing these sequencing strategies. Given the favorable safety profile and improved OS with IVO/AZA, this combination represents the preferred frontline treatment for *IDH1*m AML in patients ineligible for IC. IVO/AZA is continued indefinitely until disease progression or unacceptable toxicity with a median duration of response (DOR) of 22.1 months [10,21]. No prospective studies on discontinuation of IVO/AZA after achieving CR or after a defined number of cycles have been published to date.

**Olutasidenib,** a second-generation IDH1 inhibitor, demonstrated CR/CRh rates of 35% with a median DOR of 25.3 months and a mOS of 11.5 months, including venetoclax-exposed patients. This led to the FDA approval in December 2022 for R/R-*IDH1*m AML (not approved by the EMA) [13,22].

***IDH2*****-Mutated AML:** The AG221-AML-005 trial evaluated enasidenib/azacitidine (ENA/AZA) in ND-*IDH2*-mutated AML (*n* = 101). Despite substantially higher overall response rates (ORR) with ENA/AZA vs. AZA alone (74% vs. 36%), this did not translate into a meaningful improvement in EFS or OS; accordingly, ENA/AZA has not received FDA/EMA approval for ND disease [79]. By contrast, enasidenib is FDA-approved (Aug 2017) for R/R-*IDH2*m AML based on single-arm data, whereas the EMA application was withdrawn due to the lack of a comparator arm to establish benefit over existing treatments [76]. In the VIALE-A trial, *IDH2*m patients benefited significantly from the combination with a mOS of 27.5 vs. 13.0 months with AZA/placebo (HR 0.30, *p* < 0.001) [80]. In the ELN 2024 higher-benefit subgroup, mOS for patients with an *IDH2*m reached 36.9 months [81]. Therefore, AZA/VEN remains the preferred frontline treatment for *IDH2*m AML ineligible for IC.

**Safety profile:** IDH inhibitor-based regimens demonstrate a favorable safety profile. In AGILE, IVO/AZA was associated with lower rates of febrile neutropenia and infections compared to venetoclax-based therapy in VIALE-A (28% vs. 84% any-grade infection). Differentiation syndrome occurs in 10–20% of patients but is manageable with prompt corticosteroid initiation [4,10].

### 3.2. What Happened to Our Patient?

Due to age and comorbidities, our patient was not eligible for IC. Moreover, by ELN 2022 criteria, our patient would have been classified as an adverse risk based on *ASXL1* and *SRSF2* mutations, and the expected outcome with IC alone—without consolidation by allo-SCT—would be poor [53]. However, this risk classification was based on intensive treatment. According to the 2024 ELN recommendations, which were designed for patients not fit for IC, our patient was classified as favorable risk based on his *IDH1*m regardless of co-occurring mutations [81]. Hence, our patient was started on ivosidenib 500 mg orally daily plus azacitidine subcutaneously on days 1–7 of 28 day cycles. He tolerated treatment well with grade 2 nausea and no differentiation syndrome. Day 28 bone marrow showed a morphologic leukemia-free state (MLFS) with *IDH1* VAF decreased to 18%; by cycle 3, he achieved CRi with *IDH1* 2% VAF. At cycle 18, he remains in CR, ECOG 0, transfusion-independent, continuing IVO/AZA maintenance.

### 3.3. Emerging Frontline Combinations

Emerging data support combining IDH inhibitors with IC and with HMA/VEN-based therapy. A recent pooled analysis of 60 induction-ineligible, newly diagnosed *IDH1/2*m AML patients treated with IDH-inhibitor triplets (e.g., ivosidenib + venetoclax + azacitidine or ivosidenib/enasidenib + venetoclax + decitabine-cedazuridine) reported CRc in 92% with 2-year OS of 69%. In patients without prior HMA/VEN exposure for antecedent myelodysplastic syndrome (MDS), outcomes were stronger (2-year OS 84%) [82]. This triple combination is evaluated in the EVOLVE-1 study, an ongoing placebo-controlled, phase 3 clinical trial in which patients with ND-*IDH1*m AML or (MDS)/AML according to ICC will be randomized to either IVO/AZA with VEN or IVO/AZA with placebo [83]. Combination with 7+3 induction was tested in a phase 1 study in fit ND patients (median age 63 years), achieving CRc rates of 77% (ivosidenib) and 74% (enasidenib), with a 5-year OS of 61% and a 3-year OS of 61%, respectively [33,84]. The HOVON 150/AMLSG 29-18 has completed enrollment of 974 patients, randomizing 7+3 plus ivosidenib/enasidenib vs. placebo. For patients with co-occurring *IDH* and *FLT3* mutations, which occur in approximately 15–25% of *IDH*m AML, the combination of an IDH inhibitor with a FLT3 inhibitor represents a rational strategy. A phase 1b trial (NCT05756777) is currently evaluating gilteritinib in combination with ivosidenib (*FLT3*m/*IDH1*m) or enasidenib (*FLT3*m/*IDH2*m) in R/R-AML; efficacy results are awaited [85]. Similarly, co-occurring *IDH* and *NPM1* or *KMT2A* alterations could theoretically benefit from combined IDH and menin inhibition, though this has not yet been formally studied.

## 4. BCL-2 Inhibition in AML: Venetoclax-Based Therapies

An 81-year-old woman with a history of MDS with low blasts (MDS-LB) for three years treated with erythropoietin was diagnosed with AML after she developed progressive fatigue and petechial bleeding. Her blood counts showed WBC 1.5 × 10^9^/L, hemoglobin 8.3 g/dL, and platelets 22.10 × 10^9^/L. Bone marrow biopsy confirmed AML with ~25% myeloblasts, arising on a background of dysplasia. NGS revealed mutations in *ASXL1* (VAF ~36%) and *CEBPA* monoallelic (~3.5% VAF); cytogenetics showed a normal karyotype. She had no prior HMA therapy. By WHO 2022 criteria, her disease is classified as AML-MR based on the antecedent MDS. Per ELN 2022, she falls into the adverse-risk category due to the *ASXL1* mutation in the absence of favorable-risk markers; however, the ELN 2024 classification for HMA/VEN-treated patients would classify her as favorable risk [80,85]. Despite her advanced age, she was in a relatively good condition (ECOG 1) with well-controlled hypertension and osteoporosis.

### 4.1. What First-Line Regimen Is Recommended for This Patient?

#### 4.1.1. CPX-351 in AML with Myelodysplasia-Related Changes

CPX-351, a liposomal fixed-ratio formulation of daunorubicin and cytarabine, was approved in 2017 for therapy-related ND-AML with MDS-related changes, based on a phase 3 trial demonstrating improved mOS vs. 7+3 (9.3 vs. 5.9 months) and a durable survival benefit [16]. However, long-term follow-up confirmed that the survival benefit was largely driven by patients who proceeded to allo-SCT, whereas those who did not undergo transplant derived considerably less benefit [86]. Given our patient’s age of 81 and ECOG 1, intensive chemotherapy was not considered appropriate, and lower-intensity therapy was selected.

#### 4.1.2. Hypomethylating Agent + Venetoclax

For IC-ineligible patients and *IDH* wildtype, treatment decisions typically fall into three practical categories: (i) HMA + **venetoclax**, (ii) HMA monotherapy (azacitidine or decitabine), and (iii) best supportive care (BSC) for patients in whom disease-directed therapy is not appropriate. In the VIALE-A trial, AZA/VEN achieved a 66% CR/CRi rate and significantly prolonged mOS compared with AZA alone (14.7 vs. 9.6 months) [4]. The median DOR was 17.5 months for patients achieving CR/CRi [80]. Pivotal trial data for venetoclax-based regimens are detailed in Table 1. Across real-world studies, early death and CRc rates were comparable to VIALE-A trial results; however, OS is more heterogeneous and frequently attenuated, with mOS varying from 9.2 to 12.7 months [87]. A systematic review of 7138 patients ineligible for IC reported a pooled mOS of 10.3 months, lower than outcomes typically reported in trials [88]. In contrast, a UK NHS real-world cohort (*n* = 587) reproduced outcomes closely to VIALE-A results with a CR/CRi 67% and mOS of 13.6 months [89]. In the REVIVE prospective multicenter cohort (*n* = 209), mOS was 11.7 months. More than half had secondary AML, including patients with prior HMA exposure or MPN blast crisis who would have been ineligible for VIALE-A. When stratified by VIALE-A eligibility, mOS was 17.8 months in eligible patients (*n* = 117) vs. 10.7 months in ineligible patients (*n* = 90; *p* = 0.027), underscoring the importance of optimizing patient selection, dosing, and supportive care [90]. Venetoclax has also been combined with LDAC in VIALE-C, demonstrating a mOS benefit (8.4 vs. 4.1 months). Unlike VIALE-A, VIALE-C included 20% of patients with prior HMA exposure; OS was longer without prior HMA (8.9 months) than with prior HMA (5.6 months). This regimen is FDA-, not EMA-approved [5,91].

LDAC has also been combined with the hedgehog inhibitor **glasdegib**, which received FDA approval for AML patients ineligible for intensive chemotherapy based on a phase 2 trial showing improved CR and OS (8.8 vs. 4.9 months) [23]. However, the phase 3 BRIGHT AML 1019 trial found no survival benefit when glasdegib was added to 7+3 or azacitidine in ND-AML, questioning its role in AML therapy [92].

#### 4.1.3. Hypomethylating Agent

HMA monotherapy (azacitidine or decitabine) remains a valid option for IC-ineligible patients who cannot tolerate venetoclax [93,94]. In practice this includes azacitidine (typically SC) or decitabine (typically IV) as single agents, with BSC reserved for patients where even low-intensity therapy is not appropriate.

**Oral decitabine-cedazuridine** (DEC-C) has been approved by the EMA as monotherapy for ND-AML ineligible for IC (2023), based on the phase 3 ASCERTAIN trial demonstrating pharmacokinetic bioequivalence with IV decitabine. In the US, DEC-C is currently FDA-approved only for MDS/chronic myelomonocytic leukemia (2020). Importantly, the phase 2b ASCERTAIN-V trial, evaluating an all-oral combination of DEC-C + VEN in the frontline unfit AML setting, achieved a CRc of 63.4% and mOS of 15.5 months, with an FDA decision expected in February 2026 [48].

#### 4.1.4. Risk Stratification for HMA + Venetoclax

The ELN 2022 risk classification was developed based on outcomes of patients receiving IC and does not adequately reflect the prognosis of patients treated with HMA/VEN-based regimens. To address this, the ELN 2024 update introduced a dedicated risk classification for this patient population. Various risk stratifications (4-Gene classifier, rev.ELN2024, Mayo Clinic, and Beat AML) for lower intensity venetoclax-based therapy exist [81,93,94,95,96]. According to the ELN 2024 patients can be stratified based on the mutational status of four genes—*TP53*, *FLT3-ITD*, *NRAS*, and *KRAS*. Favorable-risk patients carry wild-type *TP53* and lack *FLT3*-ITD and *RAS* mutations; this group explicitly includes *NPM1*-, *IDH1/2*-, and *DDX41*-mutated AML but also encompasses patients with myelodysplasia-related gene mutations. Intermediate-risk patients have wild-type *TP53* but harbor *FLT3-*ITD and/or *N/KRAS* mutations, while *TP53*-mutated (*TP53*m) patients are classified as adverse risk [95]. Lachowiez et al., proposed a revised ELN 2024 classification based on a multicenter analysis of 279 patients, suggesting patients with *KRAS* mutations (mOS 3.3 months) and *PTPN11* mutations (mOS 4.9 months) be upgraded to adverse risk [93]. More recently, the Prognostic Risk Integration for Survival Modeling (PRISM) has been presented at ASH 2025 based on a multinational retrospective cohort study developed from 2273 patients [97]. A web calculator is available at prism-aml.com [98]. Importantly, the likelihood of achieving a response strongly affects survival outcomes and remains a key factor guiding treatment decisions [99].

#### 4.1.5. Safety Profile

The most clinically significant toxicity of AZA/VEN is prolonged cytopenias, particularly neutropenia. In VIALE-A, grade ≥ 3 neutropenia occurred in 42% (vs. 29% with AZA alone), and febrile neutropenia in 42% (vs. 19%) [4]. Infectious complications are frequent; antimicrobial and antifungal prophylaxis practices vary by institution and individual risk assessment. Drug interactions are a critical consideration: venetoclax is metabolized by CYP3A4, and strong CYP3A4 inhibitors—particularly azole antifungals such as posaconazole and voriconazole—require significant venetoclax dose reductions. To reduce the risk of tumor lysis syndrome, cytoreduction before starting venetoclax (target WBC < 15.10 × 10^9^/L), sufficient hydration, uricosuric prophylaxis with allopurinol, and electrolyte monitoring should be implemented [87].

#### 4.1.6. Optimal Venetoclax Schedule

The standard protocol of venetoclax combined with HMA is 400 mg daily following a ramp-up schedule of 100 mg on day 1, 200 mg on day 2, and 400 mg from day 3 onwards in each 28 day cycle. Prolonged cytopenias are the dominant practical limitation of HMA/VEN, and real-world practice commonly shortens VEN exposure (often to 21 or 14 days) after blast clearance to facilitate count recovery [100]. In the VIALE-A trial among responders who received ≥6 cycles, from cycle 6 onward, ~70% received VEN for 15–21 days per cycle and 7% for <15 days [80]. Retrospective series generally suggest that VEN 14–21 vs. 28 days can preserve remission and survival with variable reductions in treatment burden [101,102,103,104,105]. Likewise, a Mayo Clinic study of ND-AML found that shorter venetoclax schedules (14 or 21 days) achieved similar remission rates and overall survival (18.6 and 21.3 months) compared with the 28 day schedule (13.2 months) during cycle 1 [103]. Willekens et al. compared frontline AZA × 7 + VEN × 7 (“7+7”, *n* = 82) with standard HMA+VEN (*n* = 166) and found that early mortality was lower with 7+7 (8-week 6% vs. 16%), but OS was inferior in 7+7 in subsets with high expected VEN benefit (mOS 14.1 vs. 32.0 months; *p* = 0.046), highlighting the need for prospective studies to optimize VEN duration across molecular subgroups [101]. Together, prospective randomized trials are needed to define the optimal venetoclax schedule across molecular subgroups.

A central question is whether venetoclax–HMA therapy can support treatment-free remission (TFR). TFR after HMA/VEN appears achievable in a subset of ND-AML patients who stop therapy (often for intolerance), with outcomes broadly similar to those who continue azacitidine alone. Durable TFR is more likely with deeper/longer responses, particularly MRD-negative CR, favorable mutations (e.g., *NPM1*, *IDH2*), and longer treatment exposure (~≥12 cycles) [99]. Importantly, there is currently no randomized prospective evidence defining optimal post-remission strategies—including consolidation intensity, maintenance duration, or treatment discontinuation criteria—for HMA/VEN-treated patients not proceeding to allo-SCT. This represents a critical knowledge gap given the growing number of patients achieving durable remissions on venetoclax-based therapy.

### 4.2. What Happened to Our Patient?

The patient was classified as favorable risk group based on ELN 2024 and started on first-line AZA/VEN. The first two cycles were administered inpatient and complicated by profound neutropenia with infectious episodes, but CRi was achieved. To reduce recurrent cytopenia-associated infections, venetoclax exposure was shortened to 14 days per 28 day cycle with prophylactic G-CSF; after a subsequent infection, venetoclax was further reduced to 7 days/cycle. Serial bone marrow biopsies and NGS monitoring (performed after cycles 1, 3, 6, 12, and 24) demonstrated rapid blast clearance, with persistent/clonally evolving mutations—*ASXL1* remaining detectable and subsequent emergence/expansion of *TET2*-mutant subclones—despite ongoing hematological remission. She has completed 23 cycles (>2 years from diagnosis) and remains in CRh.

### 4.3. Emerging Frontline Combinations

Despite favorable initial response rates, resistance to venetoclax-based therapy remains a central challenge. Under venetoclax-selective pressure, leukemic cells can activate alternative survival pathways—including MCL-1/BCL-XL upregulation and RAS/MAPK signaling—often accompanied by monocytic differentiation that reduces BCL-2 dependence [106,107]. Beyond FLT3/IDH/menin-based triplets (described in the corresponding sections), most AML cases lack a directly actionable molecular target, making the development of further venetoclax-based combinations particularly important for this unmet need.

#### 4.3.1. Venetoclax-Based Combinations for IC-Ineligible Patients

For older patients (≥60 years) or those unfit for IC, several venetoclax-based combinations are under investigation, though none has yet been validated in randomized trials. MD Anderson evaluated cladribine + LDAC + venetoclax with consolidation alternating cladribine/LDAC/venetoclax and HMA/VEN (NCT03586609) to reduce resistance and cumulative toxicity [44,108]. The rationale for this combination is based on mechanistic synergy: cladribine, a purine analog, enhances cytarabine cytotoxicity, while venetoclax provides complementary BCL-2 inhibition. This approach achieved ~85% CRc with high MRD negativity and durable survival, including a high CR rate (~83%) in *N/KRAS*-mutant AML and particularly favorable outcomes in *NPM1-* or *DDX41*-mutated disease (mOS ~60 months) [44]. Beyond targeted small molecules, venetoclax is also being combined with novel immunotherapeutic agents in early clinical trials (Table 2). These include sabatolimab, a monoclonal antibody targeting TIM-3, a key immunoregulatory receptor involved in immune evasion; cusatuzumab, an anti-CD70 antibody targeting CD70/CD27 signaling on leukemic stem cells; the CD123-directed antibody-drug conjugate pivekimab sunirine; and CD123 × CD3 bispecific T-cell engagers such as mipletamig [46,47,50,109]. Recently, the FDA granted Breakthrough Therapy Designation for IPN60340 (ICT01, anti-BTN3A γ9δ2 T-cell activator) + AZA/VEN in first-line unfit AML, based on phase 1/2 EVICTION data with >90% of patients at the 10 mg dose achieved CRc by end of cycle 2, with a 12-month OS rate of 62% [49,110,111].

#### 4.3.2. Venetoclax-Based Combinations for IC-Eligible Patients

In younger, fit patients, venetoclax has been combined with intensive regimens such as 7+3, CLIA, and FLAG-IDA, yielding high CRc rates (85.3%, 93%, and 96%, respectively) [108,112,113]. Intensive chemotherapy has remained the treatment of choice for fit adults; however, the place of HMA/VEN in “induction-eligible” patients has been investigated in multiple retrospective studies [114]. Lu et al. demonstrated non-inferiority of decitabine/venetoclax compared to intensive 7+3 chemotherapy in a phase 2 study, further supporting the role of HMA/VEN in induction-eligible patients [115]. However, selected patients, including good-risk patients according to ELN 2022 and patients with *RUNX1*::*RUNX1T1* rearrangement, tended to benefit more from 7+3 [115]. In the prospective phase 2 PARADIGM trial, 172 induction-eligible patients were randomized to AZA/VEN vs. conventional induction (7+3 or CPX-351), focusing on an intermediate/adverse-risk population by excluding acute promyelocytic leukemia, CBF alterations, *FLT3* and *NPM1* mutations (in patients aged <60 years), *BCR::ABL1* fusion, or mixed phenotype AML. Compared to IC, AZA/VEN significantly improved the median EFS (14.6 vs. 6.2 months) with a significantly higher CRc rate (81% vs. 55%) and a greater proportion of patients proceeding to transplant (61% vs. 40%). Adverse events were primarily hematologic, occurring at similar rates between arms; grade ≥ 3 treatment-emergent infections and bleeding were ~5% lower in the AZA/VEN arm. Overall survival remains immature and was not significantly different (21.5 vs. 18.6 months, *p* = 0.18) [70]. These findings support AZA/VEN as a potential induction strategy in selected fit patients; however, longer follow-up and subgroup analyses are needed to define durability and identify those most likely to benefit. In addition, the VIALE-T trial, an ongoing randomized, open-label, phase 3 study, is investigating use of AZA/VEN in the maintenance setting after allo-SCT [116].

## 5. Menin Inhibitors—Changing the Treatment KMT2A-Rearranged and NPM1-Mutated AML

*KMT2A*-rearranged (*KMT2A*r) (~5%) and *NPM1*-mutated (*NPM1*m) (~30–40%) AML are characterized by HOX gene overexpression, which depends on the menin–KMT2A interaction [117,118]. Menin inhibitors disrupt the binding of menin to KMT2A and can reverse that process. Beyond *KMT2A* fusions, other AML subtypes, including those with *NPM1* mutations, *NUP98* rearrangements, and *UBTF* tandem duplications, similarly rely on the menin–KMT2A axis [67,117].

### 5.1. Menin Inhibitors in Clinical Development

Seven menin inhibitors—revumenib, ziftomenib, bleximenib, enzomenib, icovamenib (BMF-219), DS-1594, and BN104—have entered clinical trials, with the most advanced being revumenib and ziftomenib [119]. Revumenib received accelerated FDA approval in November 2024 for R/R-*KMT2A*r AML and, more recently (October 2025), in R/R-*NPM1*-mutated AML, demonstrating in the AUGMENT-101 trial ORR rates of 63% and 47% with median DOR of 6.4 and 4.7 months and a mOS of 8 and 4 months, respectively [18,19]. Ziftomenib was approved by the FDA in November 2025, showing an ORR of 33%, a DOR of 4.6 months, and a mOS of 6.6 months in R/R *NPM1*m AML in the phase 1b/2 KOMET-001 study (NCT04811560) [20].

### 5.2. Safety Profile

Menin inhibitors show a generally favorable safety profile, with mostly manageable, reversible toxicities. Gastrointestinal symptoms, cytopenias, and differentiation syndrome (4–16%) are most common. QT prolongation has been observed more frequently with revumenib (Grade ≥ 3 ~14–23%) [119].

### 5.3. Emerging Frontline Combinations

Menin inhibitor monotherapy can induce remissions in R/R-*KMT2Ar* or *NPM1*m AML, but responses are often not durable; consequently, combinations with IC or HMA/VEN are moving rapidly into frontline treatment. In KOMET-007, ziftomenib 600 mg in combination with IC (7+3) yielded a CRc of 94% (32/34) in ND-*NPM1*m and 83% (10/12) in ND-*KMT2A*r AML; in combination with AZA/VEN, it achieved a CRc of 84% (26/31) in ND-*NPM1*m AML [34,35]. Bleximenib 100mg + AZA/VEN showed similar signals in ALE1002 (ND: CRc 75% (*n* = 20), R/R: in VEN-naive CRc 73% (*n* = 15) and in VEN-pretreated CRc was 29% (*n* = 7)), and bleximenib + 7+3 yielded a CRc of 87.5% in ND-AML [36,37]. The SAVE trial tested the oral triplet revumenib + DEC-C + VEN, reporting a CRc of 81% (*n* = 21) and a 12-month DOR 70% in ND-AML. In the Beat AML Master Trial revumenib was combined with AZA/VEN in older ND patients (*n* = 43), demonstrating a CRc of 81% with a median DOR of 11.2 and 12 months and mOS of 18.0 and 15.5 months in *KMT2A*r and *NPM1*m patients, respectively [38,39].

Multiple phase 3 trials are now testing menin inhibitors upfront on (i) intensive-chemotherapy backbone—ziftomenib (KOMET-017-IC), bleximenib (HOVON 181 AML), and revumenib (REVEAL-ND NPM1)—or (ii) HMA/VEN backbones- ziftomenib (KOMET-017-NIC), bleximenib (cAMeLot-2), and revumenib (EVOLVE-2). These trials will clarify optimal backbones and determine whether menin inhibitor-based regimens will translate into improved event-free and overall survival. Until these data mature, menin inhibitor-based frontline combinations should be exclusively used within the frame of clinical trials.

## 6. TP53-Mutated AML: An Unresolved Challenge

*TP53*-mutated AML accounts for approximately 5–10% of newly diagnosed cases and remains the most treatment-resistant subgroup and is associated with poor outcomes observed across all available treatment modalities [120]. *TP53*m AML derives no survival benefit from venetoclax addition, despite higher response rates compared to HMA alone [121]. This outcome seems to be regardless of which HMA is used [122]. Allo-HCT is feasible in younger, fit patients; however, only a subset achieves durable control, and relapse is common. Moreover, it remains uncertain whether intensive cytotoxic induction to achieve remission is mandatory before HCT [87,120,123,124]. Several targeted strategies have been evaluated in this subgroup.

**Magrolimab** is a monoclonal antibody targeting CD47, which blocks the “don’t-eat-me” signal on leukemic cells to enhance macrophage-mediated phagocytosis [125]. Despite encouraging phase 1b/2 activity of magrolimab added to AZA/VEN in a *TP53*m subcohort, the randomized ENHANCE-3 trial was terminated early for futility, with the magrolimab arm showing more fatal adverse events (AEs) (19.0% vs. 11.4%) and inferior mOS (10.7 vs. 14.1 months) versus control [51,126]. **Eprenetapopt** (APR-246), a small molecule that restores wild-type p53 conformation, showed encouraging but preliminary phase 2 results when combined with azacitidine in AML (ORR 33%, mOS 13.9 vs. 3.0 months in patients with <30% vs. >30% bone marrow blasts, respectively [127]. However, the pivotal phase 3 trial in *TP53*m MDS failed to meet its primary CR endpoint, and development in myeloid malignancies has been placed on clinical hold. The addition of venetoclax to eprenetapopt and azacitidine has been explored in a small cohort (*n* = 30; CRc 53%, CR 37%) but has not been pursued further [128]. Given the repeated failure of single-pathway approaches, adaptive platform trials represent a rational framework for future development. The MyeloMATCH platform includes dedicated TP53-directed arms and allows systematic evaluation of novel strategies within a molecularly selected population [129]. The anti-TIM-3-directed antibody, **Sabatolimab**, was evaluated in ND AML patients in the phase II STIMULUS-AML-1 trial in combination with AZA/VEN and reported a manageable safety profile. Notably, in the *TP53*m subgroup (*n* = 21), patients achieved a CR rate of 52% and mOS of 12.6 months [45]. **Tagraxofusp**, a CD123-directed cytotoxin approved for blastic plasmacytoid dendritic cell neoplasm, was evaluated in a phase 1 study in combination with AZA ± VEN in CD123-positive AML or high-risk MDS patients, demonstrating an ORR of 54% in *TP53*m patients (*n* = 13) [130].

To date, no TP53-directed therapy has demonstrated a definitive survival benefit in randomized trials, and *TP53*m AML remains the single greatest unmet need in the field. In the absence of a suitable clinical trial, many patients with *TP53*m AML, particularly those who are older or frail, may be managed with HMA monotherapy or BSC [87].

Finally, the bone marrow microenvironment is an underappreciated driver of therapeutic resistance in *TP53*m AML. In this setting, leukemic blasts may receive pro-survival signals from stromal and immune niche components—including CXCR4-mediated retention and immunosuppressive microenvironmental remodeling—that increase resistance to both cytotoxic and targeted agents [131]. Three-dimensional bone marrow niche models and niche-like drug screening platforms that incorporate stromal, osteoblastic, and vascular components can capture microenvironment-mediated pro-survival signaling and may thereby improve the predictive accuracy of functional drug screening in AML [132].

## 7. Discussion

For decades, the treatment of AML was largely confined to IC, followed in selected cases by allo-SCT—a “one-size-fits-all” approach that did not leverage molecular therapeutic targets and was primarily applicable to younger, fitter patients [1,53]. In elderly or unfit individuals, HMAs were administered as monotherapy but achieved only modest remission rates and limited OS [133,134].

Since 2017, the therapeutic landscape of AML has changed substantially, with fourteen novel agents receiving regulatory approval (Table 1). Nevertheless, only a limited number of these agents have gained FDA and EMA approval for routine frontline use in defined AML subgroups. These include the BCL-2 inhibitor venetoclax in combination with HMA for patients unfit for IC [4]; the FLT3 inhibitors midostaurin and quizartinib in combination with IC for fit patients with *FLT3*m AML [7,9]; gemtuzumab ozogamicin combined with IC in CD33-positive AML with favorable or intermediate risk [6]; and ivosidenib plus azacitidine for IC-ineligible patients with *IDH1*m AML [10]. By contrast, agents targeting IDH2 and menin—approved in the R/R setting and demonstrating promising activity in *IDH2*- or *NPM1/KMT2A*-altered AML—are not yet available for upfront therapy, as results of ongoing randomized trials are pending (Table 2) [12,18,19].

Selection of optimal frontline therapy requires careful integration of clinical and biological factors. Age, performance status, and comorbidities determine eligibility for IC and potential candidacy for allo-SCT [135], thereby defining the therapeutic backbone (IC versus HMA-based therapy). Rapid identification of actionable targets—via flow cytometry (CD33) or molecular diagnostics (e.g., FLT3, IDH1)—is essential to enable the addition of targeted agents. However, outside clinical trials, not all rational drug combinations are currently accessible in the upfront setting. For example, FLT3 inhibitors are approved in combination with IC for fit patients, whereas IDH1 inhibitors are restricted to HMA-based regimens in IC-ineligible individuals [7,9,10].

In addition, cytogenetic and molecular abnormalities—such as *TP53* mutations or myelodysplasia-related gene alterations—as well as risk stratification according to the ELN 2022 and ELN 2024 classifications must be considered, as these substantially influence prognosis and therapeutic benefit [53,95]. Preexisting comorbidities further affect treatment choice, given the distinct toxicity profiles of the available agents (Table 1) [135]. With regard to induction backbones, IC has historically been regarded as the only potentially curative approach, whereas HMAs—even when combined with venetoclax or ivosidenib—were considered non-curative and reserved for older or frail patients [4,21]. The introduction of HMA/VEN, however, has challenged this paradigm. High response rates and a comparatively favorable safety profile have prompted evaluation of HMA/VEN in IC-eligible patients. Recent randomized trials comparing IC and HMA/VEN head-to-head in fit individuals demonstrated non-inferiority or even superiority of HMA/VEN [70,115], suggesting that this regimen may evolve into a standard of care for a broad proportion of ND-AML patients. However, PARADIGM was a phase 2 study with limited patient numbers, excluding favorable-risk patients (CBF alterations and *FLT3/NPM1* co-mutations in patients aged < 60 years), and OS data remain immature with no significant difference to date (21.5 vs. 18.6 months, *p* = 0.18). Subgroup analyses by ELN risk category, molecular profile, and transplant status have not yet been reported, and full publication is pending [70]. Confirmatory phase 3 data are needed to establish the role of HMA/VEN in IC-eligible AML.

Nonetheless, several critical questions remain. Available data indicate that certain molecularly defined subgroups, particularly favorable-risk AML according to ELN 2022, may still derive greater benefit from IC [115]. Future treatment algorithms will likely require integrative scoring systems incorporating cytogenetic, molecular, and clinical parameters to guide selection between IC and HMA/VEN. Moreover, optimal consolidation and maintenance strategies following HMA/VEN induction in patients not proceeding to allo-SCT remain undefined. It is unclear whether therapy can be safely discontinued, should be continued indefinitely, or whether IC-based consolidation might offer additional benefit as proposed by Lu et al. [115].

A key limitation of the current randomized trials is the absence of systematic integration of targeted agents into both IC and HMA/VEN arms. The combination of mutation-specific targeted therapies with either backbone represents one of the most promising strategies under active clinical investigation (Table 2). Early-phase trials suggest that adding FLT3, IDH1/2, or menin inhibitors to IC or HMA/VEN enhances response rates and may prolong OS in genetically defined subgroups [25,35,83]. Ongoing large, randomized studies (Table 2) will determine whether such combinations become standard practice, particularly given the high prevalence of *FLT3*, *IDH*, *NPM1*, and *KMT2A* alterations in AML.

The therapeutic landscape is therefore becoming increasingly complex. Clinicians will need to navigate multiple induction backbones and a growing number of targeted agents within each category. Individualized treatment decisions based on molecular profile, cytogenetics, risk stratification, age, and comorbidities will become progressively more important.

An additional area of interest is the combination of two or more targeted agents in patients harboring multiple actionable mutations (e.g., concurrent *NPM1* and *FLT3* or *IDH1* alterations). Although biologically compelling, such strategies remain investigational and are currently supported only by small, early-phase studies [85,136,137]. Key challenges include overlapping toxicities, optimal dose determination, and the question of simultaneous versus sequential administration. The definitive clinical value of multi-target combinations remains to be established in larger trials.

Despite these advances, a substantial unmet medical need persists—most notably in patients with *TP53*m AML, frequently accompanied by a complex karyotype. Five-year OS rate in this subgroup remains approximately 10–20%, and allo-SCT represents the only curative option [120]. Current strategies are largely limited to bridging approaches with IC or HMA/VEN prior to transplantation, HMA monotherapy, or BSC. Novel agents targeting CD47 or aiming to restore TP53 function (e.g., eprenetapopt) have thus far failed to demonstrate meaningful improvements in survival [41,51]. Whether emerging approaches—including microenvironment-directed therapies and immunotherapeutic strategies—can improve outcomes in *TP53*m AML remains to be determined.

## 8. Conclusions

Due to drug approvals granted in recent years in the field of AML, different therapeutic options are available for newly diagnosed patients to date. The choice between IC and HMA–based induction is currently less driven by specific molecular targets and instead primarily guided by patient-related factors such as age, comorbidities, performance status, and risk stratification according to ELN 2022 and ELN 2024 criteria. However, this paradigm may change considerably in the near future, considering the results of recent clinical trials suggesting the superiority of HMA/VEN in most patients. In selected patients, approved targeted agents are routinely added to IC or HMA and improve response rates and overall survival. However, only a limited number of all possibly synergistic drug combinations are currently approved or suitable for frontline therapy, and several treatment strategies are still being evaluated in randomized clinical trials. While outcomes may improve for patients eligible for targeted therapies, the treatment of *TP53*m AML remains a major unmet medical need. Emerging therapeutic approaches, including strategies targeting the leukemic microenvironment and immunotherapeutic concepts, require further investigation to determine their clinical impact.

## Figures and Tables

**Table 1:** FDA/EMA-approved targeted and non-targeted agents for AML since 2017.

Drug	Pivotal Trial (NCT)	Approved Indication	Key Efficacy	Safety Highlights	FDA	EMA
**FLT3 Inhibitors**						
**Midostaurin**	RATIFY [7] (NCT00651261)	ND *FLT3*m (TKD + ITD) AML, with 7+3 Ind/Consol/Maint	mOS 74.7 vs. 25.6 mo (HR 0.78)	FN, rash, mucositis, nausea	2017	2017
**Gilteritinib**	ADMIRAL [8] (NCT02421939)	R/R *FLT3*m AML, mono	mOS 9.3 vs. 5.6 mo (HR 0.64)	LFT ↑, FN, nausea	2018	2019
**Quizartinib**	QuANTUM-First [9] (NCT02668653)	ND *FLT3*-ITD+ AML, with 7+3 Ind/Consol/Maint	mOS 31.9 vs. 15.1 mo (HR 0.78)	QTc ↑, cytopenias	2023	2023
**IDH 2 Inhibitor**						
**Enasidenib**	AG221-C-001 [12] (NCT01915498)	R/R *IDH2*m AML, mono	mOS 9.3 mo; ORR 40.3%; CR 19.3%	DS, TLS, bilirubin ↑	2017	--
**IDH 1 Inhibitor**						
**Ivosidenib**	AG120-C-001 [11] (NCT02074839)	R/R *IDH1*m AML, mono	mOS 8.8 mo; CR+CRh 30.4%	DS, QTc ↑, GI events neutropenia	2018	-- ^a^
	AGILE [10,21] (NCT03173248)	ND *IDH1*m AML unfit for IC, with AZA	mOS 29.3 vs. 7.9 mo (HR 0.42); CR 47% vs. 15%		2022	2023
**Olutasidenib**	2102-02 [13,22] (NCT02719574)	R/R *IDH1*m AML, mono	mOS 11.5 mo; CR+CRh 35%	DS, nausea, fatigue	2022	--
**BCL-2 Inhibitor**						
**Venetoclax**	VIALE-A [4] (NCT02993523)	ND-AML unfit for IC, with AZA ^b^	mOS 14.7 vs. 9.6 mo (HR 0.66) CR+CRi 66.4% vs. 28.3%	Cytopenias, FN, TLS	2018	2021
	VIALE-C [5] (NCT03069352)	ND-AML unfit for IC, with LDAC ^c^	mOS 8.4 vs. 4.1 mo (HR 0.70), CR+CRi 48% vs. 13%		2018	--
**Menin Inhibitors**						
**Revumenib**	AUGMENT-101 [19] (NCT04065399)	R/R *KMT2A*r acute leukemia (adult + pediatric ≥ 1y)	mOS 8 mo ORR 63%, CR+CRh 23%,	FN, DS, QTc ↑, infections	2024	--
	AUGMENT-101 [18] (NCT04065399)	R/R *NPM1*m AML (adult + pediatric ≥ 1y)	mOS 4.8 mo, ORR 47%, CR+CRh 23.4%		2025	--
**Ziftomenib**	KO-MEN-001 [20] (NCT04067336)	R/R *NPM1*m AML (adults)	mOS 6.6 mo, ORR 33%, CR+CRh 22%	DS, FN, infections	2025	--
**Other Approved Agents**						
**Gemtuzumab ozogamicin**	ALFA-0701 [6] (NCT00927498)	ND CD33+ AML, with 7+3 IC; R/R mono (FDA only)	EFS HR 0.66 (*p* = 0.006); meta-analysis: OS benefit in fav/int risk	Hepatotoxicity, cytopenias, infusion reactions	2017	2018
**CPX-351**	301 Study [16] (NCT01696084)	ND-t-AML or AML-MRC	mOS 9.3 vs. 5.9 mo (HR 0.70)	FN, bleeding, prolonged cytopenias	2017	2018
**Glasdegib**	BRIGHT AML 1003 [23] (NCT01546038)	ND-AML unfit for IC, with LDAC	mOS 8.8 vs. 4.9 mo (HR 0.51)	Muscle spasms, QTc ↑, Dysgeusia	2018	2020
**Oral azacitidine**	QUAZAR AML-001 [15] (NCT01757535)	AML maintenance in CR1 post-IC, not transplant candidates	mOS 24.7 vs. 14.8 mo (HR 0.69)	GI events, neutropenia, FN	2020	2021
**Oral decitabine-cedazuridine**	ASCERTAIN [14,24] (NCT03306264)	ND-AML unfit for IC	bioequivalent to IV decitabine	Cytopenias, fatigue, infections	-- ^d^	2023

↑ = elevated/prolonged, ^a^ Ivosidenib monotherapy for R/R *IDH1*m AML: EMA marketing authorization application was withdrawn in 2020 due to insufficient data from the single-arm phase I study. ^b^ VIALE-A enrolled AML patients without prior HMA exposure. ^c^ VIALE-C enrolled patients with (approx. 20%) and without prior HMA exposure. ^d^ Oral decitabine-cedazuridine (DEC-C) is FDA-approved only for MDS/CMML. The combination of DEC-C + venetoclax in ND-AML (ASCERTAIN-V, NCT04657081) is under FDA review. Abbreviations are defined in Table 2 footnote.

**Table 2:** Selected frontline combination regimens for newly diagnosed AML in clinical trials.

Regimen	Trial (NCT)	Phase	Cohort °	Key Efficacy
**FLT3-Targeted Combinations**				
**Gilteritinib + AZA/VEN**	VICEROY (NCT05520567) [25]	1/2	NIC	(n = 24 [VEN400]; 44 total) CRc 91%; 12-month OS 77%
**Quizartinib + DEC/VEN**	AML-804 (NCT03661307) [26]	1/2	NIC	(n = 30) CRc 94%, CR 70%, MRD-neg 75%; mOS NR
**Midostaurin + IC ± GO ^a^**	OPTIMISE-FLT3 (ISRCTN34016918)	3 *	IC	Ph 1: (n = 77) CRc 91%; 2y OS 77% [27]
**Crenolanib + IC (7+3)**	(NCT02283177) [28]	2	IC	(n = 44) CRc 86%, CR 77%; 3y OS 71%
**Gilteritinib vs. midostaurin + IC (7+3)**	PrECOG 0905 (NCT03836209) [29]	2	IC	(n = 180) CRc: gilt 86% vs. mido 72% (*p* = 0.042); HCT 66% vs. 46%, *FLT3* clearance 83% vs. 44%
**IDH-Targeted Combinations**				
**IVO + AZA ± VEN**	EVOLVE-1 (NCT07075016)	3 *	NIC	Ph 1b/2: (n = 31) CRc 90% (IVO+VEN+AZA) vs. 83% (IVO+VEN); mOS 42 mo [30]
**DEC-C + VEN ± ENA ^b^**	MM1OA-S03 (NCT05564390) [31]	2 *	NIC	Ph 1b/2 (AML-150): IDH2m (n = 23) CRc 100%, MRD-neg 95% [32]
**IVO or ENA + IC (7+3)**	HOVON 150 (NCT03839771)	3 *	IC	Ph 1 (Stein): IVO (n = 60) CRc 77%, 12-month OS 78%, mOS NR; ENA (n = 91) CRc 74%, mOS 25.6 mo [33]
**Menin Inhibitor Combinations**				
**Ziftomenib + IC (7+3)**	KOMET-017 (NCT07007312)	3 *	IC	Ph 1 (KOMET-007): *NPM1*m (n = 34) CRc 94%, CR 88%; *KMT2A*r (n = 12) CRc 83%, CR 83% [34]
**Ziftomenib + AZA/VEN**	KOMET-017 (NCT07007312)	3 *	NIC	Ph 1 (KOMET-007): (n = 31) CRc 84%, CR 58% [35]
**Bleximenib + AZA/VEN**	cAMeLot-2 (NCT06852222)	3 *	NIC	Ph 1b (ALE1002): (n = 20) CRc 75% [36]
**Bleximenib + IC (7+3)**	HOVON 181 (NCT07223814)	3 *	IC	Ph 1b (ALE1002): (n = 24) CRc 87.5% [37]
**Revumenib + AZA/VEN**	EVOLVE-2 (NCT06652438)	2/3 *	NIC	Ph 1b (BEAT AML): (n = 43) CRc 81%, CR 67%, MRD-neg 100% (37/37) [38]
**Revumenib + DEC-C + VEN**	SAVE (NCT05360160) [39,40]	1/2	NIC	(n = 21) ORR 86%, CR 76%, CRh 5%; MRD-neg 100% (18/18)
**Revumenib + IC (7+3)**	REVEAL-ND (NCT07211958)	3 *	IC	Ph 1 (SNDX-5613-0708): (n = 26) CRc 92%, CR 69%, MRD-neg CR 86% [39,40]
**TP53-Directed Combinations**				
**Eprenetapopt + AZA/VEN**	(NCT04214860) [41]	1/2	NIC	(n = 39) CR 38%; mOS 7.3 mo; not pursued further
**Biomarker-Agnostic/Immunotherapy Combinations**				
**GO + 7+3 ± VEN ^b^**	MM1YA-A04 (NCT06917911) [42]	2 *	IC	Ph 1b (VEN+7+3): (n = 34) CRc 85%; MRD-neg 86%
**FLAG-IDA + VEN**	(NCT03214562) [43]	1/2	IC	(n = 77 [ND]; 138 total) CRc 95%, MRD-neg 90%; 3y OS 66%
**Cladribine + LDAC + VEN/AZA/VEN**	(NCT03586609) [44]	2	NIC	(n = 190) CRc 84%, CR 73%, MRD-neg 75%
**Sabatolimab (anti-TIM-3) + AZA/VEN**	STIMULUS-AML1 (NCT04150029) [45]	2	NIC	(n = 85) CR 47%; mOS 13.3 mo; MRD-neg 74% (36/49)
**Pivekimab sunirine (anti-CD123 ADC) + AZA/VEN**	IMGN632-0802 (NCT04086264) [46]	1/2	NIC	(n = 49) CRc 80%, CR 63%, MRD-neg 90%
**Mipletamig (anti-CD123) + AZA/VEN**	RAINIER (NCT05303076) [47]	1/2	NIC	(n = 13) CR 78%, MRD-neg 71% (5/7)
**DEC-C + VEN**	ASCERTAIN-V (NCT04657081) [48]	2	NIC	(n = 101) CRc 63%, CR 47%; mOS 15.5 mo
**ICT01 (anti-BTN3A) + AZA/VEN**	EVICTION (NCT04243499) [49]	1/2	NIC	(n = 57) CRc 84%, CR 68%
**Cusatuzumab (anti-CD70) + AZA/VEN**	ELEVATE (NCT04150887) [50]	1 ^b^	NIC	(n = 42) CRc 81%, CR 48%, MRD-neg 47%
**Magrolimab (anti-CD47) + AZA/VEN**	ENHANCE-3 (NCT05079230) [51]	3	NIC	(n = 378) Negative: mOS 10.7 vs. 14.1 mo (control superior)
**Tuspetinib + AZA/VEN**	TUSCANY (NCT03850574) [52]	1/2	NIC	(n = 18) CR/CRh 100%, MRD-neg 78%

° Cohort: IC = eligible for intensive chemotherapy; NIC = not eligible for intensive chemotherapy. * Listed trials are ongoing without reported results; reported key efficacy data based on a preceding study. ^a^ double-3-arm design: 7+3 + GO + mido vs. 7+3 + mido vs. FLAG-IDA + GO + mido ^b^ myeloMATCH substudy. Abbreviations (Table 1 and Table 2): ADC, antibody-drug conjugate; AML, acute myeloid leukemia; AML-MRC, AML with myelodysplasia-related changes; AZA, azacitidine; BCL-2, B-cell lymphoma 2; BTN3A, butyrophilin subfamily 3 member A; CD, cluster of differentiation; CMML, Chronic myelomonocytic leukemia; Consol, consolidation; CR, complete remission; CR1, first complete remission; CRc, composite complete remission (CR + CRh + CRi); CRh, CR with partial hematologic recovery; CRi, CR with incomplete hematologic recovery; DEC, decitabine; DEC-C, decitabine–cedazuridine; differentiation syndrome; EFS, event-free survival; ENA, enasidenib; fav/int, favorable/intermediate; FLAG-IDA, fludarabine, cytarabine, G-CSF and idarubicin; FLT3, fms-like tyrosine kinase 3; *FLT3*m, *FLT3*-mutated; FN, febrile neutropenia; GI, gastrointestinal; gilt, gilteritinib; GO, gemtuzumab ozogamicin; HCT, hematopoietic cell transplantation; HMA, hypomethylating agent; HR, hazard ratio; IC, intensive chemotherapy; IDH, isocitrate dehydrogenase; *IDH1*m, *IDH1*-mutated; *IDH2*m, *IDH2*-mutated; Ind, induction; ITD, internal tandem duplication; IV, intravenous; IVO, ivosidenib; *KMT2A*r, *KMT2A*-rearranged; LDAC, low-dose cytarabine; LFT, liver function tests; Maint, maintenance; MDS, myelodysplastic syndromes; mido, midostaurin; mOS, median overall survival; mono, monotherapy; mo, months; MRD, measurable residual disease; MRD-neg, MRD-negative; NCT, National Clinical Trial; ND, newly diagnosed; *NPM1*m, *NPM1*-mutated; NR, not reached; Ph, phase; ORR, overall response rate; OS, overall survival; R/R, relapsed/refractory; QTc, corrected QT interval; t-AML, therapy-related AML; TKD, tyrosine kinase domain; TIM-3, T-cell immunoglobulin and mucin domain-3, TLS, tumor lysis syndrome; TP53, tumor protein p53; y, years; VEN, venetoclax; 7+3, cytarabine + daunorubicin.

## Data Availability

The original contributions presented in this study are included in the article. Further inquiries can be directed to the corresponding author.

## Abbreviations

The following abbreviations are used in this manuscript:

allo-SCT	allogeneic stem cell transplantation
AE(s)	adverse event(s)
AML	acute myeloid leukemia
AML-MR	AML, myelodysplasia-related
AZA	azacitidine
ATP	adenosine triphosphate
BCL-2	B-cell lymphoma 2
BSC	best supportive care
CBF	core-binding factor
CC-486	oral azacitidine
CD	cluster of differentiation
CI	confidence interval
CPX-351	liposomal daunorubicin and cytarabine
CR	complete remission
CRc	composite complete remission
CRh	complete remission with partial hematologic recovery
CRi	complete remission with incomplete hematologic recovery
CYP3A4	cytochrome P450 3A4
CXCR4	C-X-C chemokine receptor type 4
DA	daunorubicin + cytarabine
DEC	decitabine
DEC-C	decitabine-cedazuridine
DOR	duration of response
ECOG	Eastern cooperative oncology group
EFS	event-free survival
ELN	European leukemia net
EMA	European medicines agency
ENA	enasidenib
FAB	French-American-British
FDA	food and drug administration
FLAG-IDA	fludarabine, cytarabine, G-CSF, and idarubicin
FLT3	fms-like tyrosine kinase 3
G-CSF	granulocyte colony-stimulating factor
GO	gemtuzumab ozogamicin
HCT-CI	hematopoietic cell transplantation-specific comorbidity index
HMA	hypomethylating agent
HOXHR	homeobox
HR	hazard ratio
IC	intensive chemotherapy
ICC	international consensus classification
IDH	isocitrate dehydrogenase
ITD	internal tandem duplication
IV	intravenous
IVO	ivosidenib
KMT2A	lysine methyltransferase 2A
LDAC	low-dose cytarabine
MAPK	mitogen-activated protein kinase
MCL-1	myeloid cell leukemia 1
MDS	myelodysplastic syndromes
MEK	mitogen-activated protein kinase kinase
MLFS	morphologic leukemia-free state
mOS	median overall survival
MPN	myeloproliferative neoplasm
MRD	measurable residual disease
ND	newly diagnosed
NGS	next-generation sequencing
NIC	not eligible for intensive chemotherapy
NOS	not otherwise specified
NPM1	nucleophosmin 1
ORR	overall response rate
OS	overall survival
PCR	polymerase chain reaction
PDGFR	platelet-derived growth factor receptor
PRISM	prognostic risk integration for survival modeling
QTc	corrected QT interval
R/R	relapsed/refractory
RFS	relapse-free survival
SC	subcutaneous
TFR	treatment-free remission
TIM-3	T-cell immunoglobulin and mucin-domain containing 3
TKD	tyrosine kinase domain
TP53	tumor protein p53
VAF	variant allele frequency
VEN	venetoclax
WBC	white blood cell count
WHO	world health organization
